# Cryogenic Characterisation of a Commercial Low-Noise Amplifier (LNA) for MKID Readout Systems

**DOI:** 10.3390/s26175356

**Published:** 2026-08-25

**Authors:** Dylan E. Santos-Verzilli, Diego Portero-Rodríguez, Hugo García-Vázquez, José Manuel Rodríguez Ramos, Luis Fernando Rodríguez Ramos

**Affiliations:** 1 Laboratorio de Circuitos Integrados (LABIC), Departamento de Electrónica, Área de Instrumentación, Instituto de Astrofísica de Canarias (IAC), 38205 La Laguna, Spain; 2 Departamento de Ingeniería Industrial, Universidad de La Laguna, 38206 La Laguna, Spain; 3 Departamento de Astrofísica, Universidad de La Laguna, 38206 La Laguna, Spain; 4 Wooptix, S.L., Nanotec Building, 38320 La Laguna, Spain

**Keywords:** cryogenic, vacuum, astrophysical instrumentation, integrated circuit (IC), Low-Noise Amplifier (LNA), microwaves, non-cryogenic certified components, MKIDs

## Abstract

The use of non-certified commercial electronics for cryogenic applications may be attractive due to their reduced cost and high availability, but they also carry risks related to reliability, performance, and thermal compatibility. The decision to use commercial components that are not certified for cryogenics instead of components specifically designed for such applications must be carefully weighed based on specific project needs and risk tolerances. This work presents the characterisation of a Low-Noise Amplifier (LNA) at cryogenic temperatures for use in astronomical instrumentation applications with a microwave kinetic inductance detector (MKID) readout system. The cooling system comprises a cryostat, a cold head operating in a closed-cycle helium refrigeration system based on the Gifford–McMahon principle, a compressor, connectors, cables, a vacuum pump, pressure and temperature sensors, and a temperature control system. The circuit was characterised over the temperature range of 295.4 K to 78.3 K.

## 1. Introduction

Microwave kinetic inductance detectors (MKIDs) represent an emerging type of detector that has been proven to be useful in many applications, such as particle physics, for example, in the detection of phonons [1,2] or in X-ray spectroscopy [3]. A more comprehensive review of MKIDs can be found in [4,5]. Arguably one of the most successful applications of this technology has been in the field of astrophysics. Instruments such as the MKID Exoplanet Camera for Subaru Coronagraphic Extreme Adaptive Optics (SCExAO) [6] or the DARK-speckle Near-infrared Energy-resolving Superconducting Spectrophotometer (DARKNESS) [7] at the Stellar Double Coronagraph at Palomar Observatory have been developed based on this technology.

MKIDs are superconducting resonator arrays that operate at sub-kelvin temperatures and are widely used in high-sensitivity astronomical instrumentation. Their detection mechanism is based on photon-induced changes in the surface impedance of a superconducting material. Specifically, photons with energies exceeding twice the superconducting gap energy hν>2Δ, where Δ is the superconducting energy gap, can break Cooper pairs within the superconducting film, producing quasiparticles. The increase in quasiparticle population modifies the electrodynamic properties of the superconductor, leading to measurable shifts in the resonator characteristics that can be used to detect and quantify the absorbed radiation [8,9].

**Figure 1:**
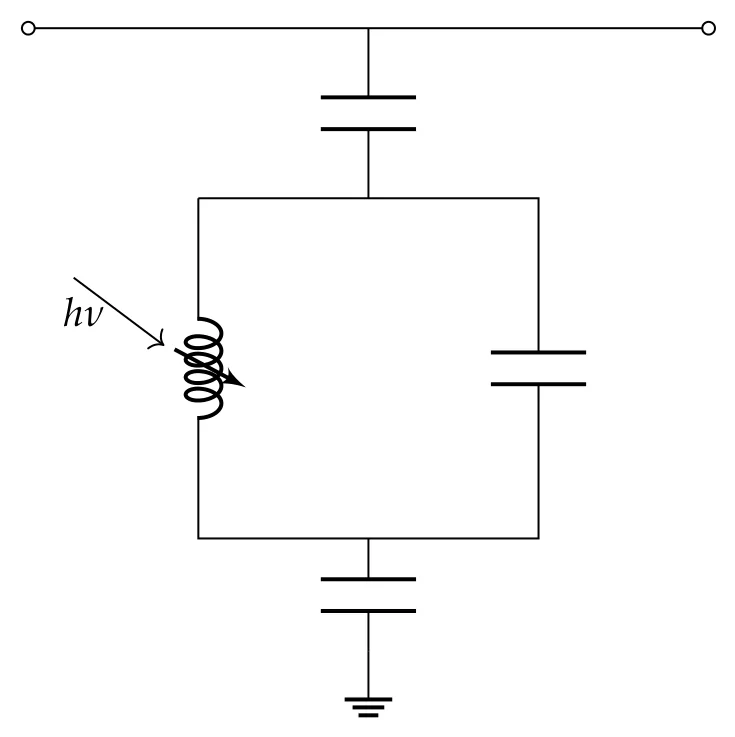
MKIDs electrical diagram as an LC resonator. An incident photon of energy hν excites the resonator mode.

An MKID is, then, a device formed by a superconducting material that has been lithographically designed in the form of an LC resonant circuit, that is, a harmonic oscillator with a defined resonance frequency, determined by its total capacitance and inductance, see Figure 1.

In astrophysical instrumentation, a wide range of electronic components are required to operate under cryogenic conditions to support highly sensitive detection systems [10,11,12,13]. However, most commercially available electronic devices are neither designed nor fully characterised for operation at such low temperatures. Manufacturers typically specify minimum operating temperatures of approximately 0 °C for commercial-grade electronics and between −40°C and −55 °C for industrial-grade devices. These temperature limits remain significantly higher than those encountered in cryogenic environments, which are generally defined as temperatures below 120K [14]. Consequently, the performance, reliability, and potential failure mechanisms of electronic components under cryogenic conditions often require dedicated characterisation and experimental evaluation prior to their deployment in such applications.

In many cases, the minimum operating temperature specified by the manufacturer does not necessarily represent the lower limit at which a component can function. Rather, it often indicates the temperature range over which the device has been characterised and qualified for commercial or industrial applications. Consequently, some commercially available components may remain operational at temperatures well below their specified limits, although their performance under such conditions is generally not guaranteed. The use of these components in cryogenic systems can offer significant cost advantages, as electronic devices specifically designed and qualified for cryogenic operation are often substantially more expensive. Therefore, the characterisation of standard commercial components at cryogenic temperatures is of considerable interest for the development of cost-effective cryogenic instrumentation.

The ZX60-83LN12+ [15] is a wideband LNA that has been characterised by the manufacturer over a frequency range between 500 MHz and 8 GHz, which is a suitable frequency range for most MKID applications. It has also been characterised for operating temperatures between −40 °C and 85°C. The designed chip operates at a supply voltage of 12 V, and the full packaging covers an area of 1.88 × 1.91 × 1.17 cm. Due to its specifications, such as its operating bandwidth (from 500 MHz to 8 GHz), gain, low noise, and high IP3, it is an attractive LNA for instrumentation projects, including applications such as radar, SATCOM, receiver front-ends, and astronomical instrumentation.

In this work, an evaluation of the ZX60-83LN12+ LNA under cryogenic conditions is performed for temperatures ranging from 295.4 K to 78.3 K. It has been characterised for frequencies ranging from 500 MHz to 8 GHz, but, in practice, it will only be used up to 2 GHz, since this is the maximum frequency at which the MKIDs used in this laboratory operate. Parameters such as gain and noise figure at different temperatures are analysed as figures of merit for the circuit’s performance under these conditions.

This work does not propose a new LNA topology or a dedicated cryogenic matching network. Instead, its contribution is the experimental evaluation of a commercially available LNA outside the temperature range specified by the manufacturer, with particular emphasis on the evolution of its gain and noise figure under cryogenic conditions. This study complements our previous characterisation of a passive attenuator [16], while addressing an active component whose gain and noise performance directly affect the overall noise of the MKID readout chain.

The paper is structured as follows: in Section 1, the MKIDs and the requirement for operation under cryogenic conditions are described, and the proposed LNA is presented. In Section 2, a description of the proposed MKIDs readout system is provided. Section 3 provides a description of the cryogenic testing platform used to perform the measurements. Section 4 presents the results obtained in this work. Finally, Section 5 concludes with the key findings of this work.

## 2. MKID Readout

**Figure 2:**
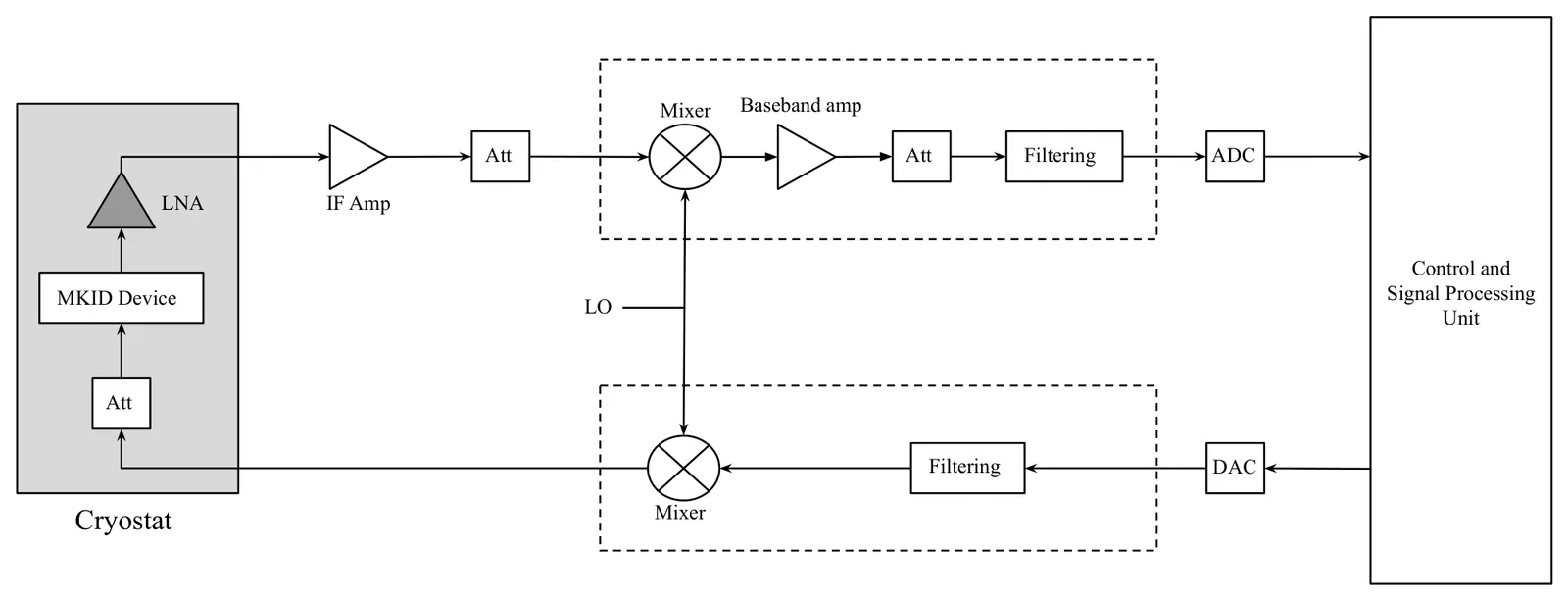
Proposed MKID readout system.

A general open-source configuration for performing the readout of these devices has already been proposed [17,18]. The proposed MKID readout system is depicted in Figure 2. In MKID-based readout systems, a control and signal-processing unit generates a frequency comb containing probe tones corresponding to the resonant frequencies of the detectors. These signals are subsequently digitised and passed through the filtering and frequency-mixing stages before being delivered to the attenuator.

The attenuator is positioned immediately upstream of the MKID array to reduce the power of the incoming signal. Although higher probe powers can improve the signal-to-noise ratio of the readout chain, excessive power dissipation can increase the temperature of the superconducting detectors, degrading their performance. Therefore, attenuation is required to ensure that the signal power reaching the MKIDs remains within acceptable limits while preserving detector sensitivity. This attenuator has already been characterised in [16]. The present work focuses on the characterisation of the Low-Noise Amplifier (LNA), an active component whose gain and noise figure directly affect the performance of the readout chain.

The main objective of placing an LNA immediately after the MKID device is to reduce the noise of the overall acquisition chain according to the Friis formula [19], Equation (1),

(1) $$NF=NF_{LNA}+\frac{NF_{rest}-1}{G_{LNA}},$$

where NF is the noise figure of the whole acquisition chain, $NF_{LNA}$ is the noise figure of the LNA, $NF_{rest}$ is the noise figure of the rest of the elements and $G_{LNA}$ is the gain of the LNA.

According to this equation, the total noise figure of the system strongly depends on the NF of the first element in the acquisition chain, in this case the LNA, while the contribution of the remaining elements is reduced by the LNA gain. Therefore, cooling the first element is highly desirable to minimise its NF without significantly affecting its gain.

For an LNA, the noise figure also provides a direct measure of the degradation of the signal-to-noise ratio (SNR). In linear units, the noise factor is defined as ($F=SNR_{in}/SNR_{out}$), while in decibels ($NF=SNR_{in},dB-SNR_{out},dB$). Therefore, the frequency- and temperature-dependent noise figure measurements presented in Section 4 directly quantify the SNR degradation introduced by the amplifier. A lower noise figure at cryogenic temperatures corresponds to a lower SNR penalty in the MKID readout chain.

## 3. Testing Platform

The cooling system comprises a cryostat equipped with a cold head operating in a closed-cycle helium refrigeration system based on the Gifford–McMahon principle [20,21], a compressor, connectors, cables, cable glands, a vacuum pump, pressure and temperature sensors, and a temperature control unit. The behaviour of the circuit was investigated over a temperature range from 295.4 K down to 78.3 K.

**Figure 3:**
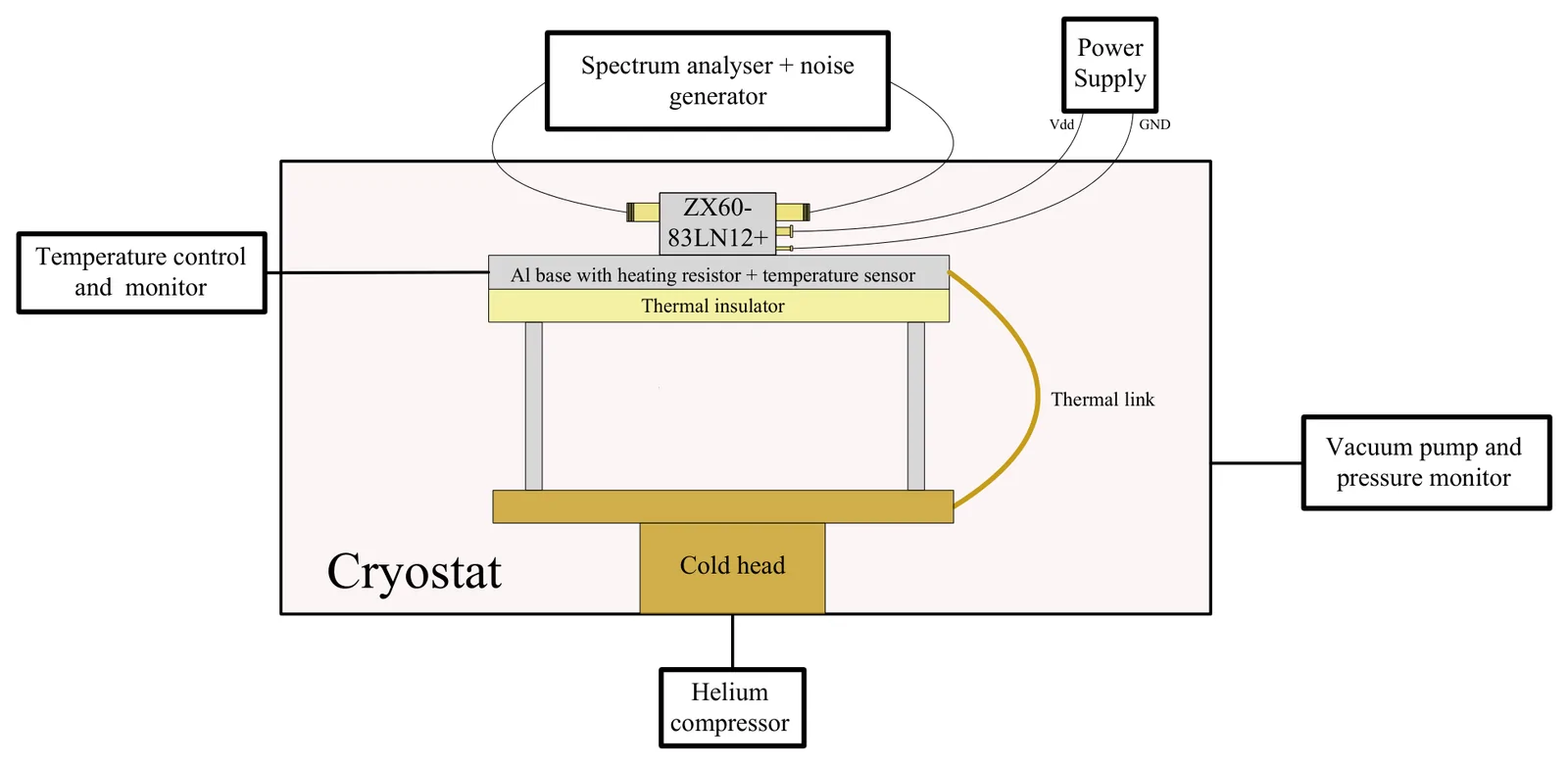
Setup used to measure the IC.

Figure 3 illustrates the experimental setup used for the LNA characterisation. Temperature regulation was achieved using a heating resistor mounted on the same surface as the LNA. To prevent potential damage to the cold head, direct heating was avoided by establishing a thermal link between the heater and the mounting surface supporting all components. A temperature sensor was positioned close to the LNA to ensure that the measured temperature closely represented the device temperature.

**Figure 4:**
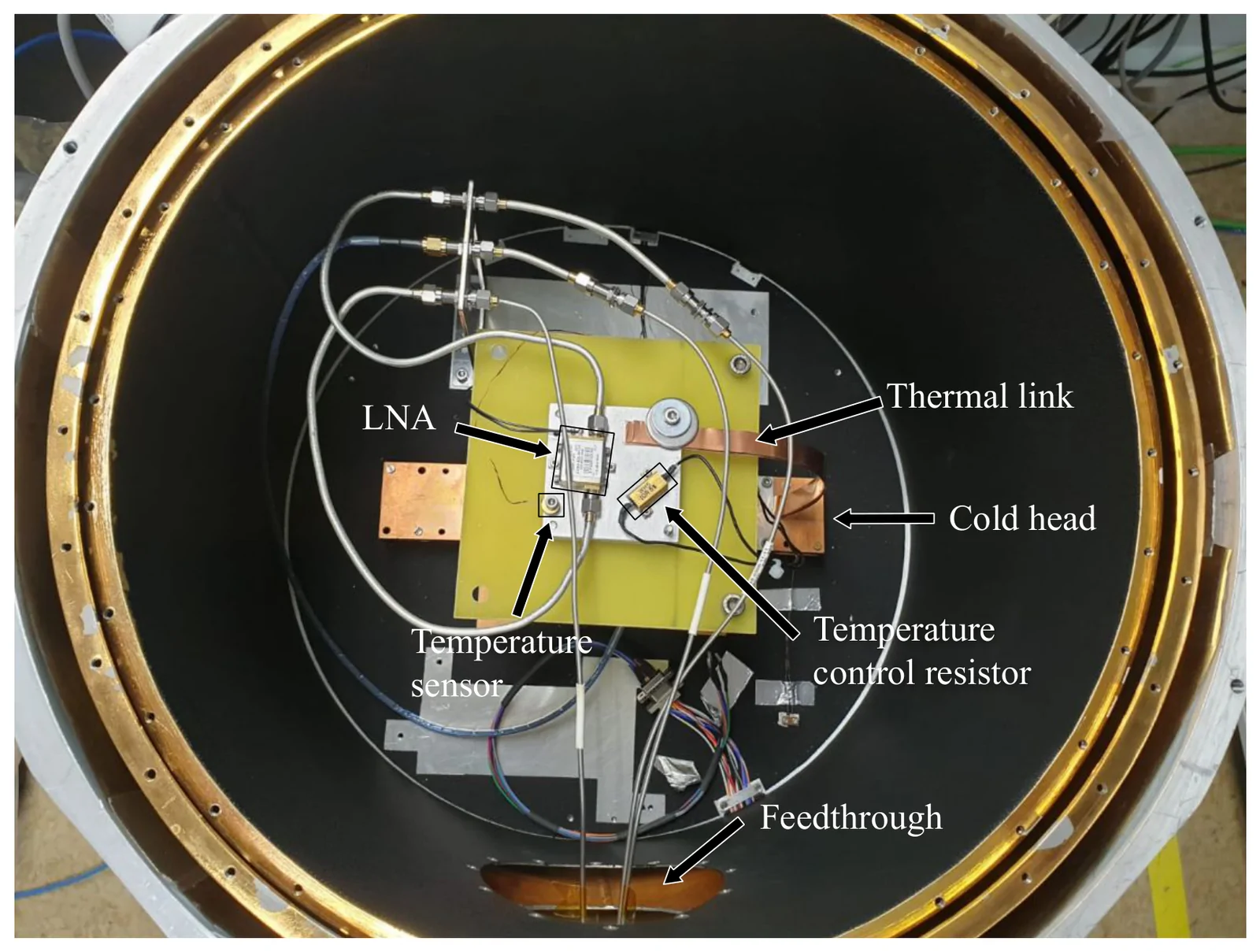
Cryostat setup.

The gain and noise figure of the circuit were measured using a spectrum analyser with a noise generator operating over a frequency range of 500 MHz to 8 GHz. Temperature monitoring and control were performed using a dedicated control system, which continuously monitored the sensor output and regulated the heating resistor accordingly. The cryostat prior to sealing for testing is shown in Figure 4.

A spectrum analyser with a noise generator was used to measure the circuit gain and noise figure over a frequency range from 500 MHz to 8 GHz. A temperature control and monitoring system was employed to track sensor readings and regulate the heating resistor. The LNA has three main connections: one input and one output with SMA connectors, and a supply connection via a coaxial cable. A semi-rigid coaxial cable (UT-141-SA-TP-M17) was used to supply the circuit during the tests performed inside the cryostat. A set of SMA connectors was then used to connect the LNA to the spectrum analyser. The insertion losses of the cables and interconnections are shown in Table 1.

## 4. Results

**Figure 5:**
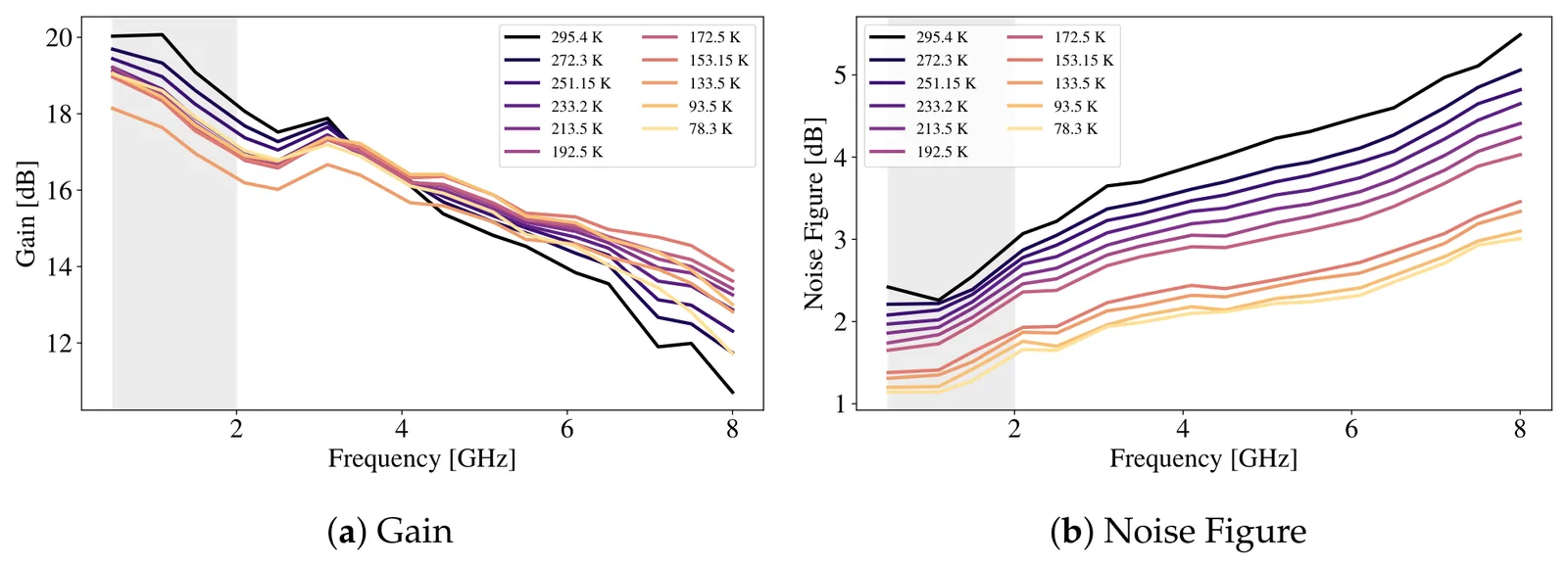
Gain and noise figure (equivalent SNR degradation) versus frequency for different temperatures.

The integrated circuit was measured under cryogenic conditions at temperatures ranging from 295.4 K (ambient temperature) to 78.3 K. The circuit was characterised within a frequency range of 500 MHz to 8 GHz. For this particular application, the frequency band of interest is between 500 MHz and 2 GHz. This is because it corresponds to the operating band of the MKIDs used by this group. Figure 5 shows the gain and noise figure across the entire frequency range.

As discussed in Section 2, the noise figure expressed in dB directly represents the SNR degradation introduced by the LNA. Therefore, Figure 5b also shows the SNR penalty as a function of frequency and temperature.

The gain decreases as the temperature decreases for frequencies below 2 GHz, with a maximum reduction of approximately 2 dB. As expected, the noise figure (NF) also decreases as the temperature decreases at all frequencies. At lower temperatures, the thermal agitation of charge carriers is reduced, lowering the internally generated noise and thus improving the noise figure. A mean improvement in the noise figure of 1.27 dB was achieved over the 0.5–2 GHz band, with individual improvements ranging from 1.12 to 1.41 dB at the three considered frequencies.

The gain reduction at cryogenic temperatures is moderate and remains below 2 dB over the frequency band of interest (500 MHz–2 GHz). Although the exact mechanism cannot be determined from the present measurements, possible contributions include a temperature-dependent shift in the amplifier bias point and changes in the impedance matching under cryogenic operation. Temperature-dependent S11 and S22 measurements were not included in the present experimental campaign; therefore, changes in the input and output matching cannot be quantitatively assessed, and the mechanisms discussed above should be regarded only as possible explanations for the observed gain variation.

In contrast, the improvement observed in the noise figure follows the expected physical behaviour of semiconductor-based low-noise amplifiers operating at reduced temperatures. Lower temperatures reduce the thermal agitation of charge carriers, decreasing the internally generated thermal noise and thereby reducing the equivalent input noise of the amplifier. From a practical perspective, this behaviour is particularly advantageous for cryogenic receiver front-ends, where the first amplification stage dominates the overall system noise according to Friis’ cascade equation. Consequently, even a moderate reduction in the LNA noise figure can produce a measurable improvement in the overall sensitivity of the receiver chain while maintaining a nearly constant gain.

Above 2 GHz, the amplifier shows lower gain and higher noise figure than in the target 0.5–2 GHz band. Nevertheless, cooling does not introduce additional degradation in this range, and no effect that could compromise the intended sub-2 GHz operation is observed.

Table 2 shows the measured gain and noise figure compared with the manufacturer specifications at room temperature and under cryogenic conditions. The losses introduced by the cables, connectors, and feedthroughs were corrected during post-processing using their characterised insertion losses. However, these losses were available at discrete frequency points and interpolation was required over the measurement band. This procedure, together with small changes in cable positioning and connector repeatability, may contribute to the residual differences between the measured room-temperature values and the datasheet specifications.

Despite the observed deviation, the same experimental setup and calibration procedure were maintained for all temperatures. Therefore, the comparison between the measurements acquired at 295 K and 78.3 K is considered representative of the temperature-dependent behaviour of the amplifier. Nevertheless, residual effects associated with the measurement setup cannot be completely excluded. For this reason, the cryogenic characterisation should be interpreted primarily in terms of the relative performance variation with temperature rather than the absolute agreement with the datasheet values.

The measured performance should also be considered in the context of LNAs specifically designed for cryogenic operation, which generally achieve lower noise levels. However, direct comparison is difficult because reported devices differ in operating temperature, frequency range, technology, and bias conditions. The aim of this work is not to demonstrate superior performance compared with dedicated cryogenic LNAs, but to evaluate the behaviour of the ZX60-83LN12+ outside its specified temperature range. At 78.3 K, the device maintains a gain of 19.04–17.03 dB and a noise figure of 1.14–1.66 dB over the 0.5–2 GHz band of interest.

## 5. Conclusions

The use of commercial electronics not certified for cryogenic operation carries some risks, but has the potential to reduce the cost of astronomical components when they are thoroughly characterised and evaluated for their potential impact on instrument performance. A large number of astronomical instruments need to operate at cryogenic temperatures. This includes not only MKID applications, but also infrared sensors and space instruments.

A cryostat was used to perform the tests. The measured frequency range extends from 500 MHz to 8 GHz, with the frequency range of interest between 500 MHz and 2 GHz. For this purpose, the losses of the cables, connectors, and feedthroughs present in the measurement system were characterised.

In this paper, the ZX60-83LN12+ LNA exhibited stable operation during the experimental campaign at temperatures down to 78.3 K, despite operating well below the minimum temperature specified by the manufacturer. While these results provide encouraging evidence of the potential suitability of this commercial RF amplifier for cryogenic front-end applications, they should not be regarded as a complete qualification for long-term cryogenic operation. A comprehensive qualification process would require additional investigations, including repeated thermal cycling, long-duration operation, and reliability assessments to evaluate possible performance degradation under realistic operating conditions. Nevertheless, the results presented here demonstrate that commercial components can represent a valuable solution for preliminary cryogenic instrumentation and prototype development. Should subsequent qualification confirm their suitability for sustained cryogenic operation, their adoption could significantly reduce the cost and complexity of MKID readout systems and other cryogenic astronomical instruments. However, the economic benefit will depend on the level of qualification required, since an extensive cryogenic qualification process may itself introduce additional costs and partially offset the cost advantage of using commercial components.

## Figures and Tables

**Table 1:** Insertion losses associated with the LNA measurement setup. (**a**) Losses at the input of the LNA. (**b**) Losses at the output of the LNA.

(**a**)				
**Frequency (GHz)**	**FLC-4FT-SMSM+**	**UT-141-SA-TP-M17 (30 cm)**	**UT-141-SA-TP-M17 (25 cm)**	**Feedthrough**
0.5	0.27 dB	0.0747 dB	0.06225 dB	0.05 dB
1	0.54 dB	0.1112 dB	0.09270 dB	0.10 dB
5	1.13 dB	0.2716 dB	0.22630 dB	0.15 dB
10	1.74 dB	0.4095 dB	0.34100 dB	0.30 dB
(**b**)				
**Frequency (GHz)**	**UT-141-SA-TP-M17 (10 cm)**	**UT-141-SA-TP-M17 (30 cm)**	**UT-141-SA-TP-M17 (25 cm)**	**Feedthrough**
0.5	0.0249 dB	0.0747 dB	0.06225 dB	0.05 dB
1	0.03707 dB	0.1112 dB	0.09270 dB	0.10 dB
5	0.09055 dB	0.2716 dB	0.22630 dB	0.15 dB
10	0.1366 dB	0.4095 dB	0.34100 dB	0.30 dB

**Table 2:** Comparison with manufacturer specifications for Gain and Noise Figure (NF).

	Gain (dB)			Noise Figure (dB)		
Frequency (GHz)	Datasheet (25 °C)	Measured (295 K)	Measured (78.3 K)	Datasheet (25 °C)	Measured (295 K)	Measured (78.3 K)
0.5	22.09	20.03	19.04	1.63	2.42	1.14
1.0	22.61	20.07	18.60	1.33	2.26	1.14
2.0	22.24	18.06	17.03	1.44	3.07	1.66
5.0	20.89	14.82	15.43	1.55	4.23	2.22
8.0	18.67	10.71	11.72	2.26	5.49	3.01

## Data Availability

The data are contained within the article. Further inquiries can be directed to the corresponding author.
